# Acute Myocardial Infarction Mortality in Young Adults in Brazil: Divergent Trends by Age, Sex, and Region, 2000-2025

**DOI:** 10.7759/cureus.117907

**Published:** 2026-10-14

**Authors:** Athos Luiz F Maia, Pedro Henrique C de Oliveira, Vittor Z Nascimento, Letícia Ribeiro S Martins, Humberto G Moreira

**Affiliations:** 1 School of Medicine, Faculdade de Medicina de Barbacena, Barbacena, BRA; 2 School of Medicine, Centro Universitário Univértix, Matipó, BRA; 3 School of Medicine, Faculdade Zarns – Itumbiara, Itumbiara, BRA; 4 School of Medicine, Faculdade Atenas de Passos, Passos, BRA; 5 School of Medicine, Federal University of Goiás, Goiânia, BRA

**Keywords:** age-standardized mortality, brazil, cardiovascular epidemiology, datasus, ecological studies, health inequalities, joinpoint regression, mortality trends, myocardial infarction, young adults

## Abstract

Background

Acute myocardial infarction (AMI) mortality has declined in Brazil, but the national figure is driven by older adults. Whether young adults have shared in that decline has not been examined.

Methods

We analyzed all deaths with AMI as the underlying cause (International Statistical Classification of Diseases and Related Health Problems, Tenth Revision (ICD-10) I21-I23) among individuals aged 20-39 years in the Brazilian Mortality Information System from 2000 to 2025. Rates were directly standardized by age and sex to the resident population enumerated in the 2022 Brazilian Demographic Census. Trends were estimated using segmented weighted log-linear (joinpoint) regression of the standardized rate, with 0-2 joinpoints selected using the Bayesian information criterion, stratified by sex, macroregion, and five-year age band. Reporting follows the REporting of studies Conducted using Observational Routinely-collected health Data (RECORD) statement.

Results

There were 59,486 deaths. The standardized rate was 4.00 per 100,000 in 2000 and 3.76 in 2025, with no net change (average annual percent change (AAPC), -0.23%; 95% CI, -0.50 to +0.04). Joinpoints occurred in 2005 and 2009: the rate declined through 2005 (annual percent change (APC), -2.64%; 95% CI, -4.33 to -0.92), rose through 2009 (+1.96%; 95% CI, +0.33 to +3.62), and then remained stable (-0.01%; 95% CI, -0.37 to +0.34). This stable national trend reflected opposing movements. Mortality rose among individuals aged 20-24 years (+3.21%; 95% CI, +2.41 to +4.01) and 25-29 years (+1.73%; 95% CI, +1.39 to +2.08) but fell among those aged 35-39 years (-0.73%; 95% CI, -0.99 to -0.46), narrowing the rate ratio for individuals aged 35-39 vs. 20-24 years from 13.1 to 6.3. Rates fell in women (-0.57%; 95% CI, -0.86 to -0.28) but not in men, widening the male-to-female ratio from 2.4 to 3.1. Regional trends ranged from -2.62% per year in the South to +1.88% in the North, reversing the regional rank order. Neither the choice of standard population nor inference robust to serial correlation altered any principal finding. Redistribution of ill-defined and nonspecific cause-of-death codes preserved the national, age, and regional patterns but eliminated the rise in the Northeast and the statistical significance of the small positive slope in men.

Conclusions

AMI mortality among young adults in Brazil stopped declining in the late 2000s. The stable national rate conceals a rising burden among adults younger than 30 years and an inverted regional gradient, neither of which is apparent in all-age surveillance. The widening sex gap points in the same direction but does not persist after correction for cause-of-death misclassification.

## Introduction

Acute myocardial infarction (AMI) is the leading single cause of death in Brazil, and national mortality from AMI has fallen over the past three decades [1-3]. That figure is used to assess Brazilian cardiovascular policy. It is also, arithmetically, a statement about older adults, who account for almost all infarctions. Whether the decline extends to young adults is a different question, and it carries different weight: a death at age 25 years represents approximately five decades of life lost, and a rising rate at that age signals an exposure that will affect the older population two or three decades from now.

Elsewhere, the findings have been concerning. Ford and Capewell found that coronary mortality among Americans aged 35-54 years had leveled off between 1980 and 2002, concealed within a falling age-adjusted total [4]. The same pattern appeared in England and Wales [5], and Wilmot et al. later described outright stagnation among Americans younger than 55 years, most pronounced in women [6]. European results have been mixed, with little evidence of plateaus concentrated in younger age groups across the European Union as a whole [7] and continued declines in the French MONICA registers [8]. Hospital surveillance shows AMI incidence rising in young women while falling in young men [9]. United States death-certificate series report reversals after 2018 [10-12], Australian linked data show attenuating declines among adults younger than 55 years [13], and the Global Burden of Disease program documents a substantial and unequally distributed burden at ages 25-49 years [14].

Brazil has not been examined at this level of detail. National analyses either cover all ages, in which the signal among young adults is numerically overshadowed [1,3], or treat age as one dimension of an age-period-cohort decomposition without isolating young adults by region [2]. Ferreira et al. showed that Brazilian trends diverge sharply by region and sex after redistribution of garbage codes, with mortality rising among men outside the capitals of the North and Northeast [1]. This finding suggests that a single national trend poorly describes the country. We are not aware of a Brazilian series restricted to ages 20-39 years, standardized within that age range, and compared across the five macroregions. This question is important because Brazil’s risk-factor trajectory differs from those of the settings in which concealed leveling was first described: tobacco use has declined while obesity and diabetes have increased, and access to reperfusion is slow and uneven [15].

We therefore estimated national and macroregional trends in age-standardized AMI mortality among Brazilian adults aged 20-39 years and identified their inflection points, stratified by sex and five-year age band. We prespecified the hypothesis that the trend in this age group would be less favorable than the decline documented at older ages.

## Materials and methods

Design and data sources

This was a nationwide ecological time-series study using routinely collected health data and reported according to the REporting of studies Conducted using Observational Routinely-collected health Data (RECORD) statement [16].

Deaths were obtained from the Mortality Information System (Sistema de Informações sobre Mortalidade, SIM) of the Brazilian Ministry of Health [17], which compiles every death certificate issued in the country and codes the underlying cause of death using the International Statistical Classification of Diseases and Related Health Problems, Tenth Revision (ICD-10) [18], which has been in continuous use in Brazil since 1996. We extracted deaths occurring between January 1, 2000, and December 31, 2025. Annual denominators came from the 2024 revision of the population projections of the Brazilian Institute of Geography and Statistics (IBGE), calibrated to the 2022 Census [19]. The standard population used for direct standardization came from a separate source, the enumerated 2022 Demographic Census [20], and is described below.

Population and case definition

We included every death of a Brazilian resident aged 20-39 years for which the underlying cause was coded as I21, I22, or I23. The three codes were analyzed together because they describe contiguous events within one index illness; using I21 alone would exclude recurrent infarction and mechanical complications. Deaths were assigned to a macroregion according to the decedent’s state of residence. The unit of analysis was the region-sex-age-band-year stratum.

The study period began in 2000, the first year for which IBGE publishes population data by age, sex, and macroregion using the current revision, and ended in 2025. Data for 2025 reflect the provisional closure of SIM and are flagged throughout.

Outcomes and standardization

The primary outcome was the age-standardized AMI mortality rate per 100,000 among adults aged 20-39 years, nationally and by macroregion. Secondary outcomes were the same rate by sex and age-specific rates in each five-year age band.

Rates were standardized using the direct method, jointly by age and sex, to the resident population enumerated in the 2022 Brazilian Demographic Census (203,080,756 persons; 62,582,094 aged 20-39 years) [20]. The standard is external to the mortality series and, deliberately, to the intercensal projections that supply the annual denominators: it is a count rather than an estimate, is fixed, and is published independently of this study. The same national standard was applied to every stratum, making all standardized rates reported here mutually comparable.

Standardization is important because the internal composition of this age group shifted: the proportion aged 20-24 years fell from 29.0% to 24.2%, while the proportion aged 35-39 years rose from 22.0% to 25.4%. Because mortality rises roughly 13-fold across this age band, crude rates would confound secular change with population aging. The correction is therefore substantial and directional, increasing the rate by 9.6% in 2000 and by 0.0% in 2022 and exceeding 3% in 15 of the 26 years. Combined-sex rates used the joint weights w(a,s) = N(a,s)/N across the eight age-by-sex cells (Appendix A); sex-specific rates used within-sex weights w(a|s) = N(a,s)/N(s) (Appendix B). This approach ensures that the identity ASR (total) = p (male) × ASR (male) + p (female) × ASR (female) holds exactly. This was verified in every year and region to within 9 × 10⁻¹⁶ per 100,000. Standardizing by age alone and applying combined-sex weights to each sex produced discrepancies of up to 0.8%. CIs were calculated using the delta method.

Because the choice of standard fixes the level of a standardized rate, we repeated the entire analysis using the same code path with two alternative standards drawn from within the series: the population in the first year (2000) and the pooled person-years from 2000 to 2025. Only the weight vector was changed (Appendix C).

Statistical analysis

Trends were modeled using segmented weighted log-linear (joinpoint) regression of the standardized rate on calendar year, with observations weighted by the inverse variance of the log rate [21]. Models with zero, one, and two joinpoints were fitted, with a minimum of five observations per segment. The best-fitting model was selected using the Bayesian information criterion. The annual percent change (APC) within a segment was calculated as (exp(β) - 1) × 100, and the average APC (AAPC) across 2000-2025 was calculated as the length-weighted geometric average of the segment slopes. A single-segment model is also reported for each stratum as a concise summary.

Six supporting analyses were prespecified. The choice of standard population was varied as described above. Pandemic-period rates were compared with values projected from the 2000-2019 trend. The primary model was refitted after excluding 2020 and 2021 and, separately, after excluding the provisional final year. Joinpoint stability was assessed using a parametric bootstrap, with 2,000 replicate series generated from the observed rates and their standard errors and refitted using the full selection procedure. A quantitative bias analysis calculated, for each principal finding, the change in AMI certification completeness between 2000 and 2025 that would be required for the trend to be entirely artifactual.

Finally, the entire analysis was repeated using a series corrected for cause-of-death misclassification. Deaths coded to ill-defined causes (R00-R99) and to the nonspecific cardiovascular codes I46, I50, I51, and I99 were extracted for the same strata and redistributed proportionally across all specific causes within each region-sex-age-band-year cell, following the approach of the Global Burden of Disease program [22]. Corrected AMI deaths were calculated as observed deaths multiplied by the ratio of all-cause deaths to specifically coded deaths in that cell. Redistributing the nonspecific cardiovascular codes across all causes rather than within circulatory diseases alone makes this correction conservative for that group. Proportional redistribution assumes that ill-defined deaths share the cause distribution of specifically coded deaths in the same cell. This assumption was applied within cells defined jointly by macroregion, sex, five-year age band, and calendar year, the finest stratification permitted by the published data. This stratification accounts for the geographic, demographic, and secular gradients along which certification quality is known to vary; the assumption is not made to hold nationally or across years. The redistributed counts were treated as observed when calculating intervals, so the intervals for the corrected estimates do not account for uncertainty from the redistribution itself and are narrower than fully propagated intervals would be. The tipping-point analysis described above addresses the same question from the opposite direction by estimating how large a differential change in certification would have to be for each finding to disappear.

Because inverse-variance weighting addresses heteroscedasticity but not temporal dependence, serial correlation was assessed in every stratum. First-order autocorrelation of the weighted residuals was tested using the Durbin-Watson statistic and a Lagrange multiplier test, in both the single-segment summary and the selected joinpoint model. The entire analysis was then repeated using heteroscedasticity- and autocorrelation-consistent (Newey-West) standard errors with a lag of 2.

Each slope was tested against the null hypothesis of no change using a two-sided Student’s t test on the regression coefficient, t = β/SE(β), with degrees of freedom equal to the number of years minus the number of estimated parameters: 24 for a single-segment model, 23 for a model with one joinpoint, and 22 for a model with two joinpoints. The t-statistic and its degrees of freedom are reported alongside every p-value in the tables. Analyses were performed using Python 3 (NumPy, SciPy, and pandas), with regression routines developed for this study and cross-validated against independent ordinary least-squares estimates. A two-sided p-value of less than 0.05 was considered statistically significant. We did not adjust for multiple comparisons because the stratified analyses were prespecified and descriptive and were interpreted based on effect sizes and interval widths.

Missing data and ethics

Age was unrecorded for 2,141 of 2,121,523 AMI deaths across all ages (0.10%), and these deaths could not be assigned to the study age band. Sex was unrecorded for 6 of the 59,486 deaths in the study age range (0.01%). Because standardization was performed jointly by age and sex, these deaths could not be assigned to a cell and were excluded from every standardized rate, which was therefore based on 59,480 deaths (99.99%). No imputation was performed.

The study used publicly available, anonymized aggregate data with no individual identifiers. Under Brazilian National Health Council Resolutions 466/2012 and 510/2016, research using such databases does not require review by a Research Ethics Committee.

## Results

Deaths and distribution

Between 2000 and 2025, there were 59,486 AMI deaths among Brazilians aged 20-39 years. Men accounted for 43,050 deaths (72.4%) and women for 16,430 (27.6%); sex was unrecorded for 6 deaths (0.01%). Table 1 presents the distribution by macroregion, which ranged from 27,247 deaths (45.8%) in the Southeast to 4,201 (7.1%) in the North and 4,231 (7.1%) in the Central-West.

**Table 1:** Deaths from AMI among adults aged 20-39 years, by macroregion and sex, Brazil, 2000-2025. Sex was unrecorded for 6 of the 59,486 deaths (0.01%). Because standardization is performed jointly by age and sex, these deaths are excluded from every standardized rate, which is therefore based on 59,480 deaths. Male and female percentages are calculated using the total number of deaths in the corresponding row as the denominator. Source: Deaths from the Mortality Information System (SIM), Brazilian Ministry of Health [17] AMI, acute myocardial infarction

Macroregion	Deaths, n (%)	Male, n (%)	Female, n (%)
Brazil	59,486 (100.0%)	43,050 (72.4%)	16,430 (27.6%)
North	4,201 (7.1%)	3,037 (72.3%)	1,164 (27.7%)
Northeast	17,129 (28.8%)	12,226 (71.4%)	4,902 (28.6%)
Southeast	27,247 (45.8%)	19,924 (73.1%)	7,319 (26.9%)
South	6,678 (11.2%)	4,765 (71.4%)	1,913 (28.6%)
Central-West	4,231 (7.1%)	3,098 (73.2%)	1,132 (26.8%)

National trend

The standardized rate was 4.00 per 100,000 in 2000 and 3.76 in 2025 (Appendix D, Figure 1). Across the study period, there was no net change, whether summarized using a single-segment APC (+0.05%, 95% CI, -0.19 to +0.28; t = 0.42, df = 24, p = 0.680) or an AAPC (-0.23%, 95% CI, -0.50 to +0.04; t = -1.78, df = 22, p = 0.088) (Tables 2, 3).

**Figure 1:**
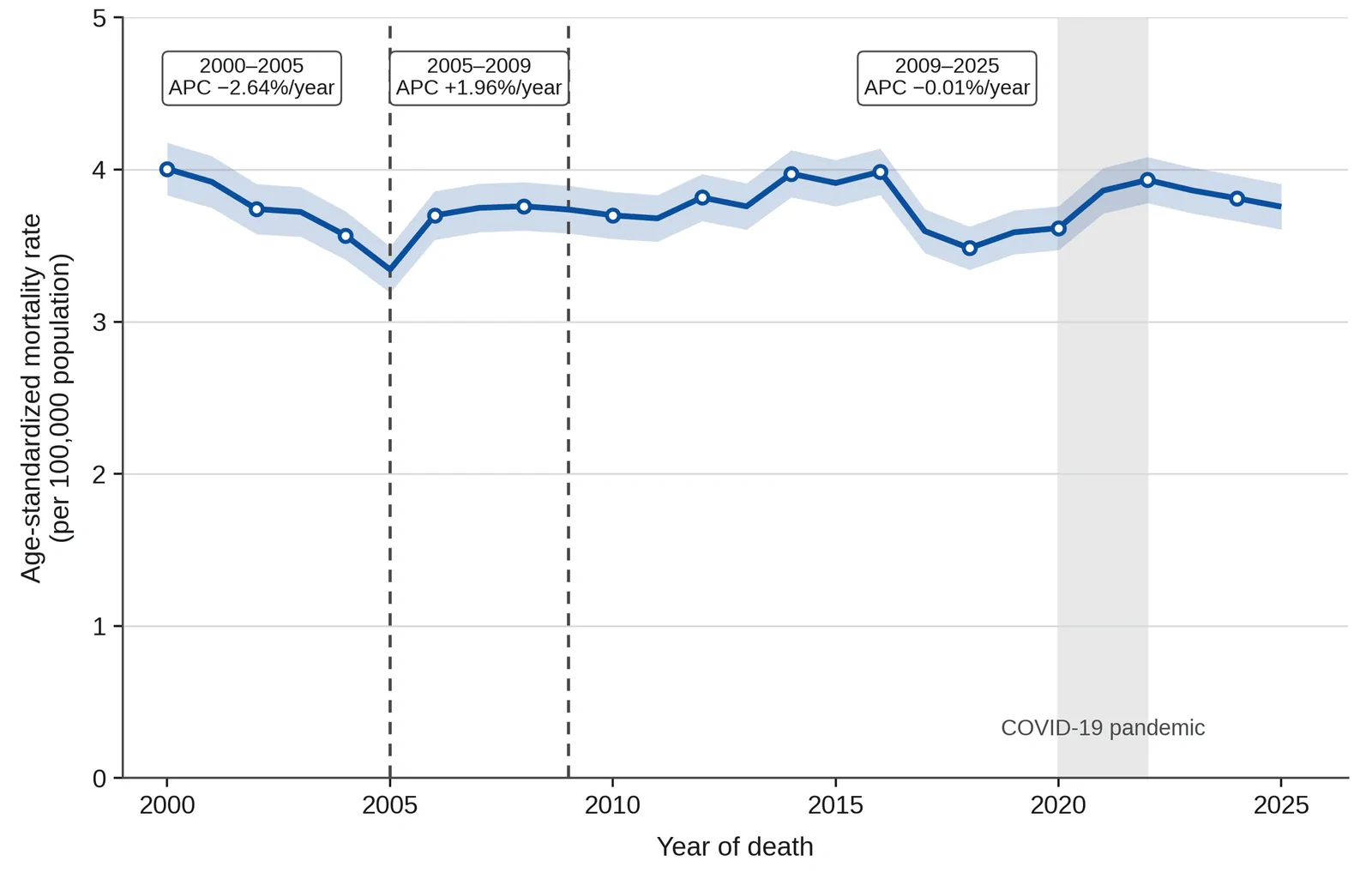
Age-standardized mortality rate from AMI among adults aged 20-39 years, Brazil, 2000-2025. Data represent the standardized rate per 100,000 population (solid line) with its 95% CI (shaded band), standardized using the direct method, jointly by age and sex, to the population enumerated in the 2022 Brazilian Demographic Census. Dashed vertical lines mark the two joinpoints, at 2005 and 2009, identified by segmented weighted log-linear regression, with the number of joinpoints selected using the Bayesian information criterion. The boxed value above each segment represents its APC. Full estimates, including CIs, t-statistics, and p-values, are provided in Table 3. Gray shading marks the COVID-19 pandemic period (2020-2022); 2025 data are preliminary. Sources: SIM/DATASUS [17] and IBGE [19,20] AMI, acute myocardial infarction; APC, annual percent change

**Table 2:** Overall change in age- and sex-standardized AMI mortality among adults aged 20-39 years, by macroregion and sex, Brazil, 2000-2025. Each slope was tested against the null hypothesis of no change using a two-sided Student’s t test on the regression coefficient (t = β/SE(β)). A two-sided p-value of less than 0.05 was considered statistically significant. All estimates have 24 degrees of freedom. Data for 2025 are preliminary (provisional SIM closure). Sources: Deaths, Mortality Information System (SIM), Brazilian Ministry of Health [17]; annual population denominators, IBGE population projections, 2024 revision [19]; standard population, 2022 Brazilian Demographic Census [20] AMI, acute myocardial infarction; APC, annual percent change

Macroregion	Sex	Deaths, n (%)	Rate 2000	Rate 2025	APC, %/year	95% CI	t	p-value
Brazil	Both sexes	59,480 (100.0%)	4	3.76	0.05	-0.19 to +0.28	0.42	0.68
	Male	43,050 (72.4%)	5.68	5.75	0.29	+0.06 to +0.52	2.58	0.016
	Female	16,430 (27.6%)	2.38	1.83	-0.57	-0.86 to -0.28	-4.00	<0.001
North	Both sexes	4,201 (7.1%)	1.96	3.32	1.32	+0.67 to +1.97	4.24	<0.001
	Male	3,037 (5.1%)	2.39	5.22	1.73	+0.99 to +2.47	4.83	<0.001
	Female	1,164 (2.0%)	1.55	1.48	0.31	-0.57 to +1.20	0.73	0.473
Northeast	Both sexes	17,128 (28.8%)	3.33	3.84	0.43	-0.17 to +1.04	1.49	0.149
	Male	12,226 (20.6%)	4.53	5.64	0.75	+0.06 to +1.44	2.26	0.033
	Female	4,902 (8.2%)	2.17	2.11	-0.34	-0.87 to +0.20	-1.30	0.206
Southeast	Both sexes	27,243 (45.8%)	4.61	4.53	0.26	-0.20 to +0.73	1.17	0.252
	Male	19,924 (33.5%)	6.73	7.11	0.48	-0.01 to +0.96	2.02	0.054
	Female	7,319 (12.3%)	2.55	2.04	-0.30	-0.81 to +0.21	-1.20	0.241
South	Both sexes	6,678 (11.2%)	4.53	2.23	-2.28	-2.72 to -1.85	-10.69	<0.001
	Male	4,765 (8.0%)	6.44	3.34	-2.19	-2.68 to -1.70	-9.19	<0.001
	Female	1,913 (3.2%)	2.69	1.16	-2.49	-3.28 to -1.69	-6.37	<0.001
Central-West	Both sexes	4,230 (7.1%)	3.38	2.86	-0.03	-0.56 to +0.50	-0.11	0.914
	Male	3,098 (5.2%)	4.71	4.37	0.22	-0.37 to +0.80	0.76	0.453
	Female	1,132 (1.9%)	2.1	1.4	-0.68	-1.66 to +0.32	-1.41	0.171

**Table 3:** Segmented (joinpoint) analysis of age- and sex-standardized AMI mortality among adults aged 20-39 years, by macroregion and sex, Brazil, 2000-2025. Each slope was tested against the null hypothesis of no change using a two-sided Student’s t test on the regression coefficient (t = β/SE(β)). A two-sided p-value of less than 0.05 was considered statistically significant. Models with two joinpoints have 22 degrees of freedom, models with one joinpoint have 23 degrees of freedom, and models with no joinpoints have 24 degrees of freedom. A single segment spanning 2000-2025 indicates that no joinpoint improved the fit. Data for 2025 are preliminary (provisional SIM closure). Sources: Deaths, Mortality Information System (SIM), Brazilian Ministry of Health [17]; annual population denominators, IBGE population projections, 2024 revision [19]; standard population, 2022 Brazilian Demographic Census [20] AAPC, average annual percent change across 2000-2025; AMI, acute myocardial infarction; APC, annual percent change within a segment

Macroregion	Sex	Segment-specific APC, %/year (95% CI)	AAPC (%/year)	95% CI	t	p-value
Brazil	Both sexes	2000-2005: -2.64 (-4.33 to -0.92); 2005-2009: +1.96 (+0.33 to +3.62); 2009-2025: -0.01 (-0.37 to +0.34)	-0.23	-0.50 to +0.04	-1.78	0.088
	Male	2000-2005: -2.20 (-3.93 to -0.43); 2005-2009: +2.16 (+0.50 to +3.85); 2009-2025: +0.21 (-0.15 to +0.57)	0.03	-0.25 to +0.31	0.22	0.825
	Female	2000-2004: -4.79 (-7.42 to -2.10); 2004-2008: +1.41 (-0.65 to +3.51); 2008-2025: -0.55 (-0.97 to -0.14)	-0.93	-1.28 to -0.59	-5.63	<0.001
North	Both sexes	2000-2009: +4.90 (+2.98 to +6.86); 2009-2025: +0.21 (-0.54 to +0.97)	1.88	+1.29 to +2.46	6.7	<0.001
	Male	2000-2009: +6.47 (+4.40 to +8.58); 2009-2025: +0.35 (-0.42 to +1.12)	2.51	+1.88 to +3.13	8.41	<0.001
	Female	2000-2021: +0.90 (-0.11 to +1.93); 2021-2025: -5.87 (-11.68 to +0.33)	-0.21	-1.18 to +0.77	-0.45	0.655
Northeast	Both sexes	2000-2010: +4.05 (+3.13 to +4.98); 2010-2025: -1.23 (-1.72 to -0.74)	0.85	+0.53 to +1.16	5.6	<0.001
	Male	2000-2011: +4.38 (+3.44 to +5.32); 2011-2025: -1.33 (-1.92 to -0.74)	1.14	+0.79 to +1.49	6.73	<0.001
	Female	2000-2010: +2.46 (+1.32 to +3.61); 2010-2021: -2.14 (-3.01 to -1.27); 2021-2025: +1.36 (-1.88 to +4.71)	0.24	-0.28 to +0.76	0.94	0.358
Southeast	Both sexes	2000-2005: -4.85 (-6.71 to -2.96); 2005-2018: +0.59 (-0.01 to +1.20); 2018-2025: +2.11 (+0.87 to +3.37)	-0.10	-0.49 to +0.29	-0.54	0.592
	Male	2000-2005: -4.43 (-6.46 to -2.36); 2005-2018: +0.61 (-0.04 to +1.27); 2018-2025: +2.74 (+1.40 to +4.09)	0.17	-0.25 to +0.59	0.83	0.413
	Female	2000-2005: -5.86 (-8.06 to -3.60); 2005-2025: +0.50 (+0.01 to +0.98)	-0.81	-1.22 to -0.40	-4.04	<0.001
South	Both sexes	2000-2005: -6.65 (-8.43 to -4.83); 2005-2025: -1.59 (-2.02 to -1.16)	-2.62	-2.96 to -2.28	-15.76	<0.001
	Male	2000-2005: -6.70 (-8.86 to -4.50); 2005-2025: -1.48 (-1.99 to -0.97)	-2.55	-2.96 to -2.14	-12.67	<0.001
	Female	2000-2025: -2.49 (-3.28 to -1.69)	-2.49	-3.28 to -1.69	-6.37	<0.001
Central-West	Both sexes	2000-2017: -0.61 (-1.42 to +0.21); 2017-2021: +4.43 (+0.84 to +8.14); 2021-2025: -4.62 (-9.17 to +0.15)	-0.48	-1.13 to +0.18	-1.52	0.144
	Male	2000-2025: +0.22 (-0.37 to +0.80)	0.22	-0.37 to +0.80	0.76	0.453
	Female	2000-2017: -2.16 (-3.55 to -0.75); 2017-2021: +9.90 (+3.30 to +16.93); 2021-2025: -9.77 (-17.51 to -1.31)	-1.61	-2.77 to -0.43	-2.82	0.01

The best-fitting model identified two inflection points, in 2005 and 2009. Mortality fell between 2000 and 2005 (APC -2.64%, 95% CI, -4.33 to -0.92; t = -3.18, p = 0.004), rose between 2005 and 2009 (+1.96%, 95% CI, +0.33 to +3.62; t = 2.50, p = 0.020), and then remained stable (-0.01%, 95% CI, -0.37 to +0.34; t = -0.07, p = 0.946; df = 22 throughout). The CI for the final segment excludes annual changes exceeding approximately 0.4% in either direction, suggesting that the absence of a trend is estimated with reasonable precision rather than simply reflecting low statistical power.

The rate level, but not the trend pattern, depended on the standard chosen (Appendix C). Standardization to the 2000 population yielded rates of 3.65 and 3.50 in 2000 and 2025, respectively, while standardization to the pooled person-years of the study period yielded rates of 3.80 and 3.61. All three specifications placed the joinpoints in 2005 and 2009 and yielded negative, nonsignificant average annual changes (-0.23%, -0.12%, and -0.16%).

Age

The flat national trend reflected movements in opposite directions across age groups (Table 4, Figure 2). Mortality rose steeply in the youngest age band, from 0.70 to 1.17 per 100,000 among adults aged 20-24 years (4,338 deaths (7.3%); APC +3.21%, 95% CI, +2.41 to +4.01; t = 8.41, p < 0.001), and also rose among those aged 25-29 years, from 1.70 to 2.39 (8,056 deaths (13.5%); APC +1.73%, 95% CI, +1.39 to +2.08; t = 10.37, p < 0.001). Mortality did not change significantly among adults aged 30-34 years (15,963 deaths (26.8%); APC +0.26%, 95% CI, -0.07 to +0.59; t = 1.60, p = 0.123) and declined among those aged 35-39 years, from 9.20 to 7.39 (31,129 deaths (52.3%); APC -0.73%, 95% CI, -0.99 to -0.46; t = -5.62, p < 0.001; df = 24 throughout). The age gradient is monotonic, with the direction of the trend reversing within a 15-year age span.

**Table 4:** Age-specific AMI mortality within the 20-to-39-year age group, by five-year age band, Brazil, 2000-2025. Each slope was tested against the null hypothesis of no change using a two-sided Student’s t test on the regression coefficient (t = β/SE(β)). A two-sided p-value of less than 0.05 was considered statistically significant. The ratio of the mortality rate at ages 35-39 years to that at ages 20-24 years fell from 13.1 in 2000 to 6.3 in 2025. Sources: Deaths, Mortality Information System (SIM), Brazilian Ministry of Health [17]; annual population denominators, IBGE population projections, 2024 revision [19]; standard population, 2022 Brazilian Demographic Census [20] AAPC, average annual percent change from the selected joinpoint model; AMI, acute myocardial infarction; APC, annual percent change from a single-segment model (df = 24)

Age band	Deaths, n	Rate 2000	Rate 2025	APC (%/year)	95% CI	p	Joinpoints	AAPC (95% CI)
20-24 years	4,338	0.7	1.17	3.21	+2.41 to +4.01	<0.001	2010; 2015	+2.43 (+1.85 to +3.01)
25-29 years	8,056	1.7	2.39	1.73	+1.39 to +2.08	<0.001	2010; 2014	+1.43 (+1.07 to +1.80)
30-34 years	15,963	4.2	4.03	0.26	-0.07 to +0.59	0.123	-	+0.26 (-0.07 to +0.59)
35-39 years	31,129	9.2	7.39	-0.73	-0.99 to -0.46	<0.001	2005; 2009	-1.02 (-1.32 to -0.72)

**Figure 2:**
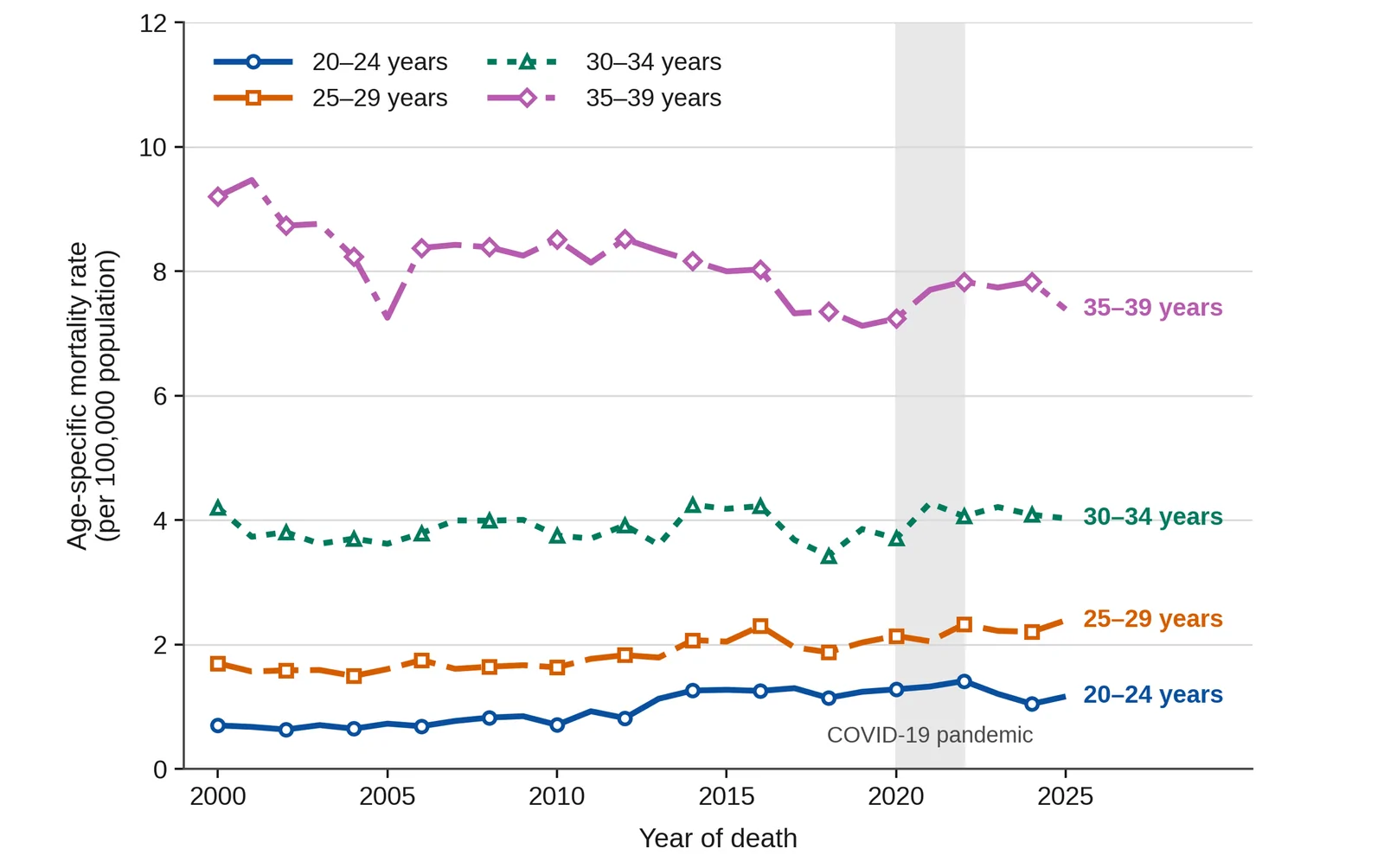
Age-specific mortality rate from AMI by five-year age band within the 20-39-year group, Brazil, 2000-2025. Data are presented as crude, age-specific rates per 100,000 population within the corresponding age band, with both sexes combined. Because each five-year age band is already homogeneous in age, these rates are not standardized. Each of the four bands is distinguished by a different combination of color, line style, and marker shape. Each series is labeled directly at its right-hand end and in the legend. Trend estimates for the four series, each obtained using weighted log-linear regression and tested with a two-sided Student’s t test on the regression coefficient (df = 24), are reported with their t-statistics and p-values in Table 4. A two-sided p-value of less than 0.05 was considered statistically significant. The shaded vertical band marks the COVID-19 pandemic period (2020-2022). The vertical axis begins at zero. Sources: SIM/DATASUS [17] and IBGE [19] AMI, acute myocardial infarction

The age structure of the burden shifted accordingly. In 2000, the mortality rate at ages 35-39 years was 13.1 times that at ages 20-24 years; by 2025, this ratio had fallen to 6.3. The oldest age band still accounted for most deaths, with 31,129 of the 59,486 deaths.

Sex

Trends also diverged by sex (Table 2, Figure 3). In men, the rate barely changed, from 5.68 in 2000 to 5.75 in 2025, with a small positive slope (APC +0.29%, 95% CI +0.06 to +0.52; t = 2.58, df = 24, p = 0.016) and an AAPC indistinguishable from zero (+0.03%, 95% CI -0.25 to +0.31; p = 0.825). In women, the rate fell from 2.38 to 1.83 (APC -0.57%, 95% CI -0.86 to -0.28; t = -4.00, df = 24, p < 0.001). The male-to-female ratio widened from 2.4 to 3.1.

**Figure 3:**
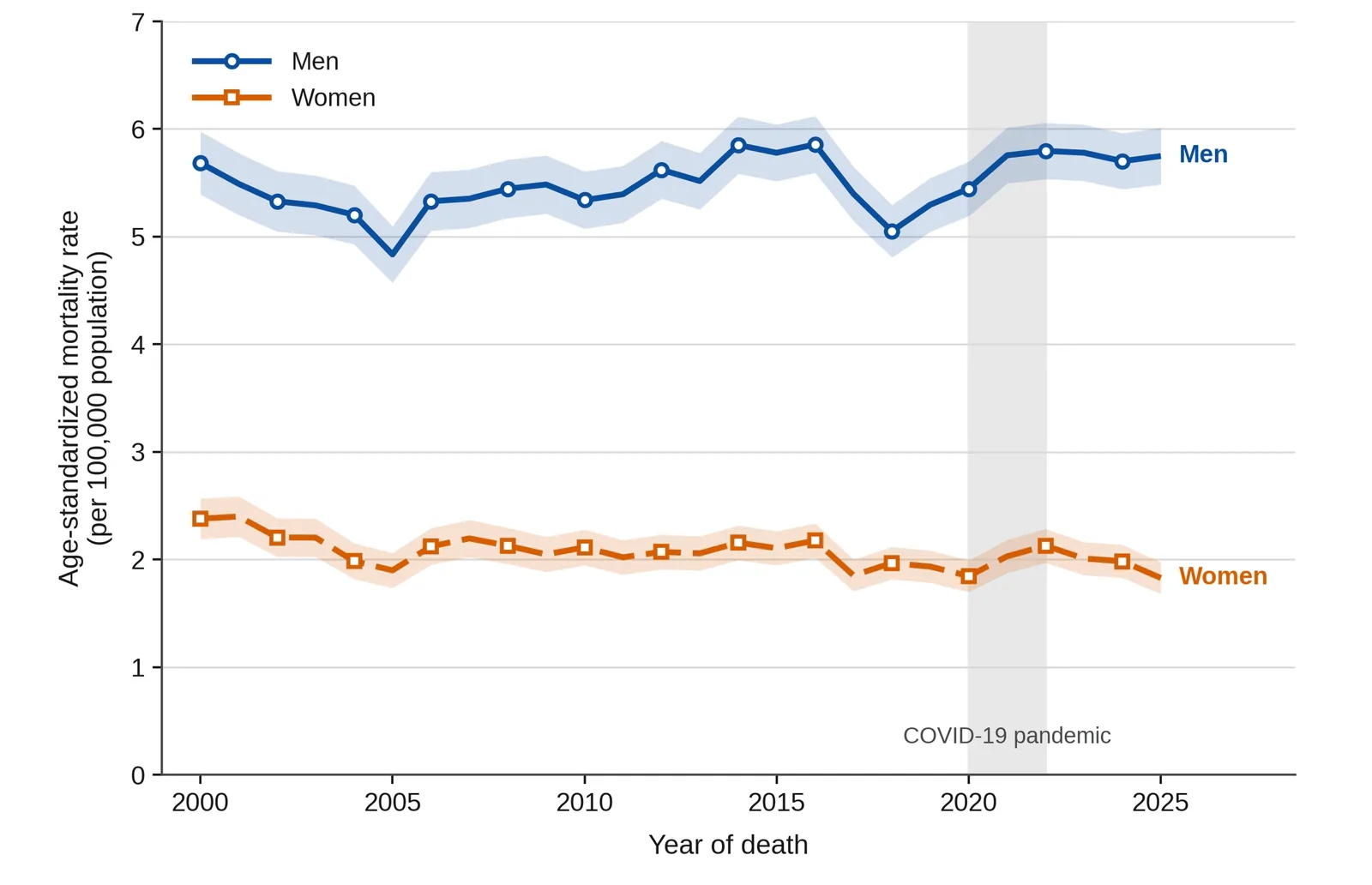
Age-standardized mortality rate from AMI among adults aged 20-39 years, by sex, Brazil, 2000-2025. Data are standardized rates per 100,000 population, with 95% CIs (shaded bands). Men are shown as a solid line with circular markers, and women as a dashed line with square markers; each series is labeled at its right-hand end. Each sex was standardized to the age distribution of the same sex in the 2022 Census, so the two series decompose exactly into the combined rate shown in Figure 1. Trend estimates, with CIs, t-statistics, and p-values, are given in Table 2. Gray shading marks the COVID-19 pandemic period (2020-2022); 2025 data are preliminary. Sources: SIM/DATASUS [17] and IBGE [19,20] AMI, acute myocardial infarction

Macroregions

Regional trends spanned the full range from sustained decline to sustained increase (Tables 2, 3, Figure 4A-4E). The South declined throughout (Figure 4D; AAPC -2.62%, 95% CI -2.96 to -2.28; t = -15.76, p < 0.001), with its rate halving from 4.53 to 2.23, while the North rose from 1.96 to 3.32 (Figure 4A; AAPC +1.88%, 95% CI +1.29 to +2.46; t = 6.70, p < 0.001), with the increase concentrated before 2009 and rates remaining flat thereafter. The Northeast rose until 2010 and then declined (Figure 4B), resulting in a net AAPC of +0.85% (95% CI +0.53 to +1.16; t = 5.60, p < 0.001). The Southeast showed no net change (Figure 4C; AAPC -0.10%, 95% CI -0.49 to +0.29; t = -0.54, p = 0.592), but its rate rose at an accelerating pace from 2018 (+2.11%, 95% CI +0.87 to +3.37; t = 3.53, p = 0.002). The Central-West showed no significant change (Figure 4E; AAPC -0.48%, 95% CI -1.13 to +0.18; t = -1.52, p = 0.144).

**Figure 4:**
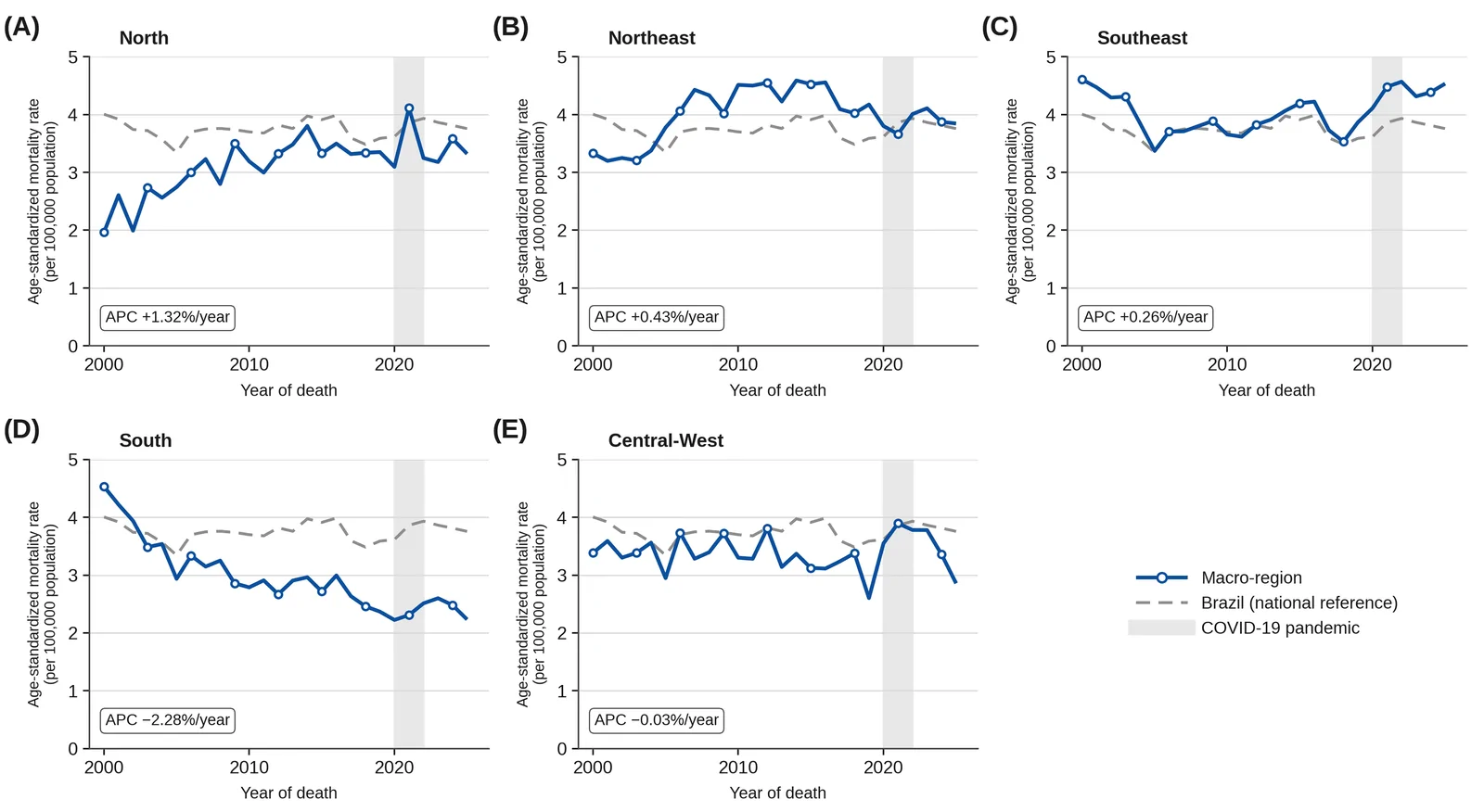
Age- and sex-standardized mortality rate from AMI among adults aged 20-39 years, by macroregion, Brazil, 2000-2025. (A) North, (B) Northeast, (C) Southeast, (D) South, and (E) Central-West. Data are standardized rates per 100,000 population. Each panel shows one macroregion as a solid line with circular markers, with the national rate repeated as a gray dashed line for comparison; the key occupies the sixth cell of the grid. All panels share the same vertical scale, so panel heights are directly comparable. The boxed value in each panel is the APC over the period, estimated using single-segment weighted log-linear regression; the segmented-model estimates, with CIs, t-statistics, and p-values, are given in Table 3. Gray shading marks the COVID-19 pandemic period (2020-2022); 2025 data are preliminary. Sources: SIM/DATASUS [17] and IBGE [19,20] AMI, acute myocardial infarction; APC, annual percent change

These movements reordered the hierarchy rather than compressing it. In 2000, the South had the second-highest rate, and the North had the lowest; by 2025, the South had the lowest rate, and the North had moved above the Central-West. The highest-to-lowest regional ratio barely changed, from 2.3-fold to 2.0-fold: the inequality persisted while its geography inverted.

Pandemic period, robustness, and bias

Against a pre-pandemic trend that was itself flat (APC -0.08%, 95% CI -0.46 to +0.31; t = -0.43, df = 18, p = 0.674), observed rates were 2.6% below the projected values in 2020, then 4.2% above in 2021, 6.1% above in 2022, and 4.4%, 3.0%, and 1.6% above in 2023, 2024, and 2025, respectively (Appendix E).

Excluding 2020 and 2021 left the model unchanged (AAPC -0.22%, 95% CI -0.50 to +0.06), as did excluding the provisional final year (AAPC -0.24%, 95% CI -0.53 to +0.05) (Appendix F). The bootstrap analysis distinguished stable findings from less stable ones (Appendix G): the joinpoint years varied across replicates, with the second joinpoint having a median of 2009 and a 95% range of 2008 to 2019. The final segment was nonsignificant in 1,787 of 2,000 replicates, and a two-joinpoint model was selected in 1,189 replicates. The joinpoint years should therefore be interpreted as approximate; the absence of a later change is more robust.

The bias analysis quantified the extent of misclassification required to explain the observed patterns (Appendix H). To eliminate the divergence between the 20-24 and 35-39 age bands, certification completeness would have had to improve 165% more in the younger band than in the older band within the same regions and years. The regional findings were more susceptible to bias: a 39% relative improvement in certification completeness could account for the increase in the North.

Redistribution further clarified the potential impact of ill-defined causes of death. Ill-defined and nonspecific codes accounted for 10.1% of all deaths among people aged 20-39 years in 2000 and 6.3% in 2025. The decline was steepest in the regions where it was expected to be greatest: from 16.5% to 6.0% in the North and from 16.1% to 5.0% in the Northeast, compared with 5.0% to 4.9% in the South (Appendix I). Correction therefore increased estimates for earlier years more than those for later years and increased northern rates more than southern rates, raising the overall death count by 8.1%, from 59,480 to 64,309 (Appendix J).

The national, age-specific, and regional patterns persisted after correction, which shifted the national series away from an increase rather than toward one: the corrected APC was -0.18% (95% CI -0.42 to +0.07), and the corrected AAPC was -0.46% (95% CI -0.70 to -0.21). The age gradient was unchanged and marginally steeper (+3.14%, +1.62%, +0.06%, and -0.97% across the four bands), because nonspecific coding was more common in the older bands and declined faster there. The South’s decline was unaffected (-2.34%, 95% CI -2.77 to -1.90), and the male-to-female ratio still widened, from 2.3 to 3.2. Three estimates changed materially: the increase in the North attenuated by about one-third but remained significant (+0.86%, 95% CI +0.30 to +1.42); the Northeast’s increase disappeared (-0.00%, 95% CI -0.46 to +0.46); and the small positive slope among men was no longer significant (+0.11%, 95% CI -0.13 to +0.35). The Northeast trend and the male trend are therefore sensitive to how ill-defined deaths are handled and are reported as such throughout.

Serial correlation behaved as a feature of model specification rather than of the data (Appendix K). In the single-segment summaries, first-order autocorrelation of the weighted residuals was detectable in 11 of the 22 strata (median Durbin-Watson statistic, 1.20). In the selected joinpoint models, it was detectable in 4 strata, two of which had negative coefficients, and the median Durbin-Watson statistic rose to 1.89. Thus, the segmented specification absorbed most of the temporal dependence left by a straight-line model. Modest positive dependence persisted in the two national series: both sexes combined (ρ = 0.44, p = 0.029) and men (ρ = 0.46, p = 0.021). Newey-West standard errors inflated the model-based standard errors by a factor of 0.78 to 1.60 (median, 1.25) and left every principal finding unchanged: the national null result remained nonsignificant (p = 0.680 to p = 0.736), the decline in the South and the increase in the North remained significant at p < 0.01, and the increase at ages 20-24 years and the decline at ages 35-39 years remained significant at p < 0.001. One subgroup estimate changed significance status: the male trend in the Northeast (p = 0.033 to p = 0.146), which was also one of the two estimates sensitive to cause-of-death coding.

Age ranges outside the study population

Over the same period, using the same source and method, mortality fell by 35.1% among people aged 40-64 years (AAPC -1.61%, 95% CI -1.77 to -1.46; t = -20.95, p < 0.001) and by 25.1% among those aged 65 years and older (AAPC -1.06%, 95% CI -1.20 to -0.91; t = -15.09, p < 0.001) (Appendix L). The 40-44-year age band followed a pattern similar to that of the older population, with a 35.2% decline, and contributed roughly half of all deaths among people younger than 45 years. The composite 20-44-year series therefore declined (AAPC -1.00%, 95% CI -1.21 to -0.79; t = -9.97, p < 0.001).

## Discussion

AMI mortality among Brazilian adults aged 20-39 years stopped falling in the late 2000s and has not resumed its decline. The national trend is flat, but it does not describe every group within the population. Mortality rose in the youngest age band and fell in the oldest, declined in women but not in men, and the regional hierarchy inverted, with the South halving its rate while the North moved in the opposite direction. Regional inequality was not reduced over this period; it was relocated.

The plateau in Brazil is an instance of the concealed leveling described by Ford and Capewell [4] and O’Flaherty et al. [5], and further characterized by Wilmot et al. as evidence of stagnation below middle age [6]. Two features extend that literature. The first is the setting: an upper-middle-income country with a universal health system and a different risk-factor history, which argues against explanations based solely on health-system dynamics in high-income countries. The second is recursion. The concealment repeats at a lower level, since even within a 20-year age window, the aggregate trend is flat while the youngest stratum shows an increase. Concealed leveling is not a property of broad age categories; it reappears at whatever level of detail the analysis stops.

The shape of the age gradient narrows, without resolving, the possible explanations. A uniform deterioration in acute care would affect all four age bands similarly and would not, by itself, produce a monotonic gradient that changes sign within a 15-year span. However, access, diagnostic practices, time to reperfusion, and case fatality need not change uniformly across ages, and an age-differentiated change in any of these factors remains a candidate explanation that these data cannot exclude. A cohort-based explanation fits the shape of the gradient at least as well. Successive Brazilian cohorts reach adulthood with a heavier cardiometabolic burden, with morbid obesity in early adulthood having risen steeply over the past two decades [23], while the decline in smoking decelerated and then reversed in the same age range [24]. The balance of these exposures plausibly differs by age, with declining tobacco exposure favoring strata in which that risk had already accumulated and rising adiposity affecting cohorts exposed from childhood. Santos et al. reached a compatible conclusion using age-period-cohort modeling [2]. Our study design cannot test this explanation, and the cohort account remains a hypothesis consistent with the gradient rather than a demonstrated pathway.

The divergence by sex runs counter to reports from the United States, where stagnation among young adults has been most pronounced in women [6,9]. Two cautions apply. The mechanism is unclear, and this is the least robust of the principal findings: the male slope did not persist after redistribution of ill-defined codes, and a modest differential change in certification completeness between the sexes would be sufficient to account for it. We therefore describe a divergence in trends, not an increase in mortality among men.

The regional inversion has the greatest policy relevance and the least mechanistic support, and it is the finding we tested most rigorously. Improved death certification in the North and Northeast was the obvious competing explanation, and redistribution of ill-defined codes eliminated the increase in the Northeast entirely and attenuated the increase in the North. What remains is an increase in the North that persisted after the correction designed to eliminate it.

The geographic pattern points toward access rather than exposure. In a single state served by one hospital able to offer primary angioplasty to patients in the public health system, the VICTIM registry found that fewer than half of patients with ST-elevation myocardial infarction received this treatment, fibrinolysis was used in a small minority, and the median time from symptom onset to arrival at that hospital was measured in hours rather than minutes [15]. Ferreira et al. found that the contrast between state capitals and the rest of the country was sufficiently wide that macroregional averages understate it [1]. A myocardial infarction in a young adult is more survivable than one in an older adult when treated promptly. Thus, a region where time to treatment is long may convert the same incidence into more deaths. Mortality data alone cannot test this hypothesis, and we do not claim to have done so. We note only that it is the explanation most consistent with a gradient that follows the geography of health services rather than that of risk factors.

Mortality in this age group has not returned to its pre-pandemic level. Rates were above the projected trend from the second year of the pandemic onward and remained above it at the end of the series. Leite et al. reported a comparable pattern for ischemic heart disease across all ages in Brazil, with the earlier decline decelerating and excess mortality persisting after the acute phase [25]. Across 16 countries, excess cardiovascular mortality also continued into the second year of the pandemic and tracked its peaks [26]. Our study design cannot separate the direct effects of infection from those of disrupted care or delayed presentation, and the excess is small relative to the uncertainty around the trend. Nevertheless, it has not closed, which is a reason to continue reporting it rather than absorb it into a period effect.

The upper limit of the age window determines the conclusion. Over the same period, using the same source and method, mortality fell substantially among people aged 40-64 years and those aged 65 years and older. Thus, the plateau is not a national feature but a characteristic of this age group. Much of the comparable literature defines young adulthood as younger than 45 years, and in these data, that convention is decisive rather than cosmetic: the 40-44-year band behaves like the older population and contributes about half of all deaths among people younger than 45 years, so the composite series declines and the plateau disappears. The same source and method, with a five-year change in the upper age limit, yield opposite conclusions. Narrow age bands make the signal visible, and this may help explain why stagnation has been reported in some settings but not others. For the same reason, projections of continued decline based on all-age data by Siqueira and de Souza [3] should not be extended to young adults, among whom the decline had already stopped within the age range used to fit those projections.

The implication for surveillance is narrow and clear. Brazilian cardiovascular targets are monitored using all-age mortality, which continues to fall and continues to indicate progress. However, this measure has been insensitive for more than a decade without progress in this age group and active deterioration in its youngest stratum. Reporting young adults as an explicit stratum would make this pattern visible at negligible cost, since the data already exist in the national mortality system [17]. We do not consider these data sufficient to justify changes in individual clinical practice, such as lowering the age at which risk stratification begins. Such changes would require evidence on incidence, risk-factor distribution, and treatment effects in this population, none of which can be supplied by an ecological analysis of mortality.

The strengths of the study are its completeness and duration: a national census of death certificates covering 26 consecutive years, with rates standardized by age and sex against an external enumerated reference population and shown to be insensitive to that choice. The trend model was selected on the basis of fit rather than assumed, and the principal threat to validity was addressed through correction rather than merely discussed.

Limitations

Four limitations bound the interpretation. Misclassification of cause of death is the most consequential, and it is the limitation we addressed rather than assumed away; its effect on every principal estimate is reported in the Results. Redistribution is nevertheless a model, not a measurement. It assumes that ill-defined deaths are distributed like specifically coded deaths in the same stratum, spreads nonspecific cardiovascular codes across all causes rather than only within circulatory diseases, and treats redistributed counts as observed when calculating CIs. Consequently, it underestimates the true uncertainty around the corrected estimates by an amount we have not quantified. Two findings depend on this correction: the increase in the Northeast and the small positive slope in men. Neither is presented here as secure. Second, this is an ecological analysis of population rates. It cannot support causal inference, and the risk-factor and access mechanisms discussed above are drawn from external sources with no linkage to these deaths. Third, residual positive serial correlation persisted in the two national series after segmentation. Although inference robust to this correlation did not change any conclusions, the model-based CIs for those series are likely to be marginally narrow. Finally, the annual denominators are intercensal projections except in census years, although the standard population used to weight the rates is based on enumerated counts. In addition, macroregions are internally heterogeneous. The substantial contrast between state capitals and the rest of the country reported by Ferreira et al. [1] is averaged out at this scale, so inequality within regions is likely to exceed the differences reported here.

## Conclusions

AMI mortality among Brazilian adults aged 20-39 years fell until the mid-2000s and has remained flat since. The stable national rate conceals a rising burden among adults younger than 30 years and an inverted regional gradient, both of which persisted after correction for cause-of-death misclassification, as well as a widening sex gap that did not. Surveillance that reports young adults as a distinct stratum is needed if Brazilian cardiovascular policy is to detect a trend that all-age indicators do not register.

## Appendices

Appendix A 

**Table 5:** Standard population and joint age-by-sex weights used for direct standardization of the combined-sex rates. ASR = Σ w(a,s) × r(a,s) × 100,000 over all eight age-by-sex cells; weights sum to 1.000000. Sex shares of the standard are p(male) = 0.491505 and p(female) = 0.508495. Standardizing by age and sex jointly makes the identity ASR(total) = p(male) × ASR(male) + p(female) × ASR(female) hold exactly in every year and region; it was verified to within 9 × 10⁻¹⁶ per 100,000. Source: 2022 Brazilian Demographic Census, resident population by sex and five-year age group (IBGE, SIDRA table 9514) [20]

Age band	Sex	Standard population, n	Weight w(a,s)	Share (%)
20–24 years	Male	7,767,306	0.124114	12.411
20–24 years	Female	7,699,157	0.123025	12.302
25–29 years	Male	7,627,458	0.121879	12.188
25–29 years	Female	7,842,265	0.125312	12.531
30–34 years	Male	7,537,285	0.120438	12.044
30–34 years	Female	7,935,832	0.126807	12.681
35–39 years	Male	7,827,333	0.125073	12.507
35–39 years	Female	8,345,458	0.133352	13.335
Total (20–39 years)	—	62,582,094	1.00000	100

Appendix B  

**Table 6:** Within-sex age weights used for the sex-specific standardized rates. w(a | s) = N(a,s) / N(s). Using within-sex rather than combined-sex weights is what makes the male and female rates decompose exactly into the combined rate of Appendix A; weighting both sexes by the combined-sex distribution breaks that decomposition by up to 0.8%. Source: 2022 Brazilian Demographic Census, resident population by sex and five-year age group (IBGE, SIDRA table 9514) [20]

Age band	Male population, n	Weight w(a | male)	Female population, n	Weight w(a | female)
20–24 years	7,767,306	0.252518	7,699,157	0.241939
25–29 years	7,627,458	0.247972	7,842,265	0.246436
30–34 years	7,537,285	0.24504	7,935,832	0.249376
35–39 years	7,827,333	0.25447	8,345,458	0.262248
Total	30,759,382	1.00000	31,822,712	1.00000

Appendix C 

**Table 7:** Sensitivity of the national age- and sex-standardized trend to the choice of standard population, Brazil, 2000–2025. Each slope was tested against the null of no change with a two-sided Student's t test on the regression coefficient (t = β/SE(β)). Two-sided p < 0.05 was considered statistically significant. All three models have df = 22. All three specifications select joinpoints at 2005 and 2009 and return a negative, nonsignificant average annual change. The 2022 Census standard is the primary analysis because it is an enumerated count external to the series; the other two are drawn from within it and are shown for comparison only. AAPC, average annual percent change Sources: Deaths, Mortality Information System (SIM), Brazilian Ministry of Health [17]; annual population denominators, IBGE population projections, 2024 revision [19]; standard population, 2022 Brazilian Demographic Census [20]

Standard population	Rate 2000	Rate 2025	AAPC (%/year)	95% CI	Joinpoints
Census 2022 (primary, external)	4.00	3.76	−0.23	−0.50 to +0.04	2005; 2009
First year of the series (2000)	3.65	3.50	−0.12	−0.39 to +0.15	2005; 2009
Pooled person-years 2000-2025	3.80	3.61	−0.16	−0.43 to +0.11	2005; 2009

Appendix D 

**Table 8:** Age- and sex-standardized acute myocardial infarction mortality rate by macroregion and sex, every year, Brazil, 2000–2025. Sources: deaths, Mortality Information System (SIM), Brazilian Ministry of Health [17]; annual population denominators, IBGE population projections, 2024 revision [19]; standard population, 2022 Brazilian Demographic Census [20]

Macroregion	Sex	2000	2001	2002	2003	2004	2005	2006	2007	2008	2009	2010	2011	2012	2013	2014	2015	2016	2017	2018	2019	2020	2021	2022	2023	2024	2025
Brazil	Both sexes	4.00 (3.83–4.18)	3.92 (3.75–4.09)	3.74 (3.58–3.91)	3.72 (3.56–3.88)	3.57 (3.41–3.73)	3.34 (3.19–3.50)	3.70 (3.54–3.86)	3.75 (3.59–3.91)	3.76 (3.60–3.92)	3.74 (3.58–3.89)	3.70 (3.54–3.85)	3.68 (3.53–3.83)	3.82 (3.66–3.97)	3.76 (3.61–3.91)	3.97 (3.82–4.13)	3.91 (3.76–4.06)	3.99 (3.83–4.14)	3.60 (3.45–3.74)	3.48 (3.34–3.63)	3.59 (3.44–3.73)	3.62 (3.47–3.76)	3.86 (3.71–4.01)	3.93 (3.78–4.08)	3.86 (3.71–4.01)	3.81 (3.66–3.96)	3.76 (3.61–3.91)
	Male	5.68 (5.39–5.98)	5.49 (5.20–5.78)	5.33 (5.05–5.61)	5.29 (5.01–5.57)	5.20 (4.93–5.48)	4.83 (4.57–5.10)	5.33 (5.06–5.60)	5.35 (5.08–5.62)	5.45 (5.17–5.72)	5.48 (5.21–5.76)	5.34 (5.07–5.61)	5.39 (5.13–5.66)	5.62 (5.35–5.89)	5.52 (5.25–5.78)	5.85 (5.58–6.12)	5.78 (5.52–6.04)	5.86 (5.59–6.12)	5.40 (5.15–5.65)	5.05 (4.81–5.29)	5.30 (5.05–5.55)	5.45 (5.19–5.70)	5.76 (5.50–6.02)	5.80 (5.54–6.06)	5.78 (5.52–6.04)	5.70 (5.44–5.96)	5.75 (5.49–6.01)
	Female	2.38 (2.19–2.57)	2.40 (2.21–2.59)	2.21 (2.03–2.38)	2.20 (2.03–2.38)	1.99 (1.82–2.15)	1.90 (1.74–2.06)	2.12 (1.95–2.29)	2.20 (2.02–2.37)	2.13 (1.96–2.30)	2.05 (1.89–2.21)	2.11 (1.95–2.28)	2.02 (1.86–2.18)	2.07 (1.91–2.23)	2.06 (1.90–2.22)	2.16 (2.00–2.32)	2.11 (1.95–2.26)	2.18 (2.02–2.34)	1.85 (1.71–2.00)	1.97 (1.82–2.12)	1.94 (1.79–2.08)	1.85 (1.70–1.99)	2.03 (1.88–2.18)	2.13 (1.97–2.29)	2.01 (1.86–2.16)	1.98 (1.83–2.14)	1.83 (1.68–1.98)
North	Both sexes	1.96 (1.49–2.43)	2.61 (2.08–3.13)	1.99 (1.54–2.45)	2.74 (2.21–3.26)	2.56 (2.06–3.06)	2.74 (2.24–3.25)	3.00 (2.47–3.52)	3.23 (2.69–3.77)	2.80 (2.31–3.29)	3.50 (2.97–4.03)	3.19 (2.68–3.70)	3.00 (2.51–3.48)	3.32 (2.82–3.82)	3.48 (2.97–3.99)	3.80 (3.28–4.32)	3.33 (2.85–3.81)	3.50 (3.01–3.98)	3.32 (2.84–3.79)	3.34 (2.87–3.81)	3.35 (2.88–3.82)	3.09 (2.64–3.55)	4.11 (3.60–4.63)	3.25 (2.79–3.71)	3.18 (2.72–3.63)	3.58 (3.10–4.06)	3.32 (2.86–3.78)
	Male	2.39 (1.67–3.10)	3.05 (2.27–3.83)	2.85 (2.09–3.61)	3.73 (2.88–4.59)	3.61 (2.76–4.45)	4.00 (3.15–4.85)	4.34 (3.45–5.24)	4.36 (3.47–5.25)	3.77 (2.98–4.56)	5.36 (4.42–6.29)	5.02 (4.12–5.93)	4.34 (3.51–5.16)	4.70 (3.85–5.54)	5.04 (4.18–5.89)	5.29 (4.43–6.16)	4.89 (4.06–5.71)	5.05 (4.22–5.88)	5.13 (4.30–5.97)	4.98 (4.16–5.79)	4.64 (3.86–5.43)	4.36 (3.61–5.12)	6.08 (5.18–6.97)	4.77 (3.98–5.56)	4.90 (4.10–5.70)	5.25 (4.43–6.08)	5.22 (4.40–6.05)
	Female	1.55 (0.95–2.16)	2.18 (1.47–2.88)	1.16 (0.66–1.67)	1.77 (1.15–2.39)	1.55 (1.01–2.09)	1.53 (0.98–2.08)	1.70 (1.13–2.26)	2.13 (1.51–2.75)	1.86 (1.28–2.43)	1.71 (1.18–2.24)	1.42 (0.94–1.89)	1.70 (1.18–2.22)	2.00 (1.45–2.54)	1.98 (1.43–2.53)	2.36 (1.78–2.95)	1.82 (1.31–2.33)	2.00 (1.47–2.52)	1.56 (1.10–2.02)	1.75 (1.27–2.24)	2.10 (1.57–2.63)	1.87 (1.37–2.37)	2.22 (1.67–2.76)	1.77 (1.29–2.26)	1.52 (1.07–1.96)	1.96 (1.46–2.47)	1.48 (1.04–1.92)
Northeast	Both sexes	3.33 (3.02–3.64)	3.20 (2.90–3.50)	3.25 (2.95–3.55)	3.20 (2.91–3.50)	3.37 (3.07–3.68)	3.78 (3.46–4.09)	4.06 (3.73–4.38)	4.43 (4.09–4.76)	4.33 (4.00–4.66)	4.02 (3.70–4.33)	4.51 (4.18–4.84)	4.50 (4.17–4.83)	4.55 (4.22–4.88)	4.22 (3.91–4.54)	4.59 (4.27–4.91)	4.52 (4.21–4.84)	4.56 (4.24–4.87)	4.09 (3.80–4.39)	4.02 (3.73–4.32)	4.17 (3.87–4.47)	3.80 (3.52–4.09)	3.66 (3.38–3.94)	4.01 (3.71–4.30)	4.11 (3.81–4.41)	3.87 (3.58–4.16)	3.84 (3.55–4.14)
	Male	4.53 (4.01–5.04)	4.36 (3.85–4.86)	4.47 (3.97–4.97)	4.32 (3.83–4.82)	4.73 (4.22–5.24)	5.21 (4.69–5.74)	5.74 (5.19–6.29)	6.41 (5.83–6.99)	6.22 (5.65–6.78)	5.62 (5.08–6.15)	6.51 (5.94–7.08)	6.68 (6.11–7.25)	6.55 (5.99–7.11)	6.15 (5.62–6.69)	6.81 (6.25–7.36)	6.90 (6.34–7.45)	6.68 (6.14–7.23)	6.15 (5.63–6.66)	5.65 (5.15–6.14)	6.27 (5.75–6.80)	5.68 (5.18–6.18)	5.58 (5.09–6.07)	5.98 (5.47–6.49)	5.90 (5.39–6.41)	5.80 (5.29–6.31)	5.64 (5.14–6.14)
	Female	2.17 (1.82–2.52)	2.08 (1.74–2.42)	2.07 (1.73–2.41)	2.12 (1.78–2.46)	2.07 (1.73–2.40)	2.39 (2.03–2.74)	2.43 (2.08–2.79)	2.51 (2.15–2.86)	2.51 (2.16–2.86)	2.48 (2.13–2.82)	2.58 (2.23–2.93)	2.39 (2.05–2.72)	2.61 (2.26–2.96)	2.36 (2.03–2.69)	2.44 (2.11–2.77)	2.23 (1.92–2.54)	2.50 (2.17–2.82)	2.11 (1.81–2.40)	2.45 (2.13–2.77)	2.14 (1.84–2.44)	1.98 (1.70–2.27)	1.81 (1.53–2.08)	2.10 (1.80–2.40)	2.37 (2.05–2.69)	2.01 (1.72–2.30)	2.11 (1.81–2.41)
Southeast	Both sexes	4.61 (4.33–4.88)	4.47 (4.20–4.74)	4.29 (4.03–4.56)	4.31 (4.04–4.57)	3.85 (3.60–4.10)	3.37 (3.14–3.60)	3.71 (3.46–3.95)	3.71 (3.47–3.95)	3.80 (3.56–4.04)	3.89 (3.65–4.13)	3.65 (3.42–3.89)	3.62 (3.38–3.85)	3.82 (3.59–4.06)	3.91 (3.67–4.15)	4.07 (3.83–4.30)	4.19 (3.95–4.44)	4.22 (3.98–4.46)	3.73 (3.50–3.96)	3.53 (3.31–3.75)	3.87 (3.63–4.10)	4.11 (3.87–4.35)	4.48 (4.23–4.73)	4.57 (4.31–4.82)	4.31 (4.07–4.56)	4.38 (4.13–4.63)	4.53 (4.28–4.79)
	Male	6.73 (6.25–7.21)	6.40 (5.93–6.86)	6.20 (5.74–6.65)	6.18 (5.73–6.64)	5.88 (5.44–6.32)	5.00 (4.60–5.40)	5.46 (5.04–5.88)	5.38 (4.97–5.79)	5.64 (5.22–6.06)	5.76 (5.34–6.18)	5.27 (4.87–5.67)	5.20 (4.81–5.60)	5.67 (5.26–6.08)	5.85 (5.44–6.26)	6.04 (5.62–6.45)	6.24 (5.82–6.66)	6.24 (5.82–6.66)	5.54 (5.15–5.94)	5.16 (4.78–5.54)	5.65 (5.25–6.05)	6.36 (5.94–6.79)	6.62 (6.19–7.06)	6.89 (6.45–7.33)	6.64 (6.20–7.08)	6.55 (6.11–6.99)	7.11 (6.65–7.56)
	Female	2.55 (2.26–2.84)	2.61 (2.32–2.90)	2.45 (2.17–2.74)	2.50 (2.21–2.78)	1.88 (1.64–2.13)	1.80 (1.56–2.03)	2.01 (1.76–2.26)	2.09 (1.84–2.35)	2.02 (1.77–2.27)	2.08 (1.83–2.33)	2.09 (1.84–2.34)	2.08 (1.84–2.33)	2.04 (1.80–2.28)	2.04 (1.80–2.28)	2.16 (1.91–2.40)	2.22 (1.97–2.47)	2.27 (2.02–2.52)	1.97 (1.74–2.21)	1.95 (1.72–2.18)	2.14 (1.90–2.39)	1.93 (1.70–2.16)	2.40 (2.15–2.66)	2.32 (2.06–2.58)	2.06 (1.82–2.31)	2.29 (2.03–2.55)	2.04 (1.80–2.29)
South	Both sexes	4.53 (4.07–5.00)	4.22 (3.77–4.67)	3.94 (3.51–4.37)	3.48 (3.08–3.89)	3.54 (3.13–3.95)	2.94 (2.56–3.31)	3.34 (2.94–3.73)	3.15 (2.76–3.53)	3.25 (2.86–3.64)	2.86 (2.49–3.22)	2.79 (2.43–3.15)	2.91 (2.55–3.27)	2.67 (2.32–3.02)	2.91 (2.55–3.26)	2.96 (2.60–3.32)	2.72 (2.38–3.06)	2.99 (2.64–3.35)	2.64 (2.31–2.97)	2.46 (2.14–2.78)	2.37 (2.06–2.68)	2.22 (1.92–2.53)	2.31 (2.00–2.62)	2.51 (2.19–2.83)	2.60 (2.27–2.92)	2.48 (2.16–2.80)	2.23 (1.93–2.53)
	Male	6.44 (5.65–7.23)	6.00 (5.24–6.76)	5.67 (4.93–6.40)	5.07 (4.37–5.77)	5.04 (4.34–5.73)	4.43 (3.78–5.08)	4.69 (4.02–5.36)	4.16 (3.53–4.78)	4.71 (4.04–5.37)	4.41 (3.77–5.06)	3.43 (2.87–4.00)	4.34 (3.71–4.97)	4.02 (3.42–4.63)	4.03 (3.43–4.63)	4.33 (3.71–4.94)	3.48 (2.93–4.03)	4.46 (3.85–5.07)	3.96 (3.38–4.53)	3.52 (2.98–4.06)	3.61 (3.07–4.16)	3.37 (2.84–3.89)	3.34 (2.82–3.86)	3.38 (2.85–3.90)	3.69 (3.14–4.23)	3.69 (3.14–4.24)	3.34 (2.82–3.86)
	Female	2.69 (2.18–3.20)	2.50 (2.01–2.98)	2.27 (1.81–2.73)	1.95 (1.52–2.38)	2.09 (1.65–2.53)	1.49 (1.12–1.87)	2.02 (1.59–2.46)	2.17 (1.72–2.62)	1.84 (1.43–2.26)	1.35 (1.00–1.71)	2.17 (1.72–2.61)	1.53 (1.15–1.90)	1.36 (1.01–1.71)	1.81 (1.41–2.22)	1.64 (1.26–2.02)	1.98 (1.57–2.39)	1.58 (1.21–1.94)	1.36 (1.02–1.70)	1.44 (1.09–1.78)	1.16 (0.85–1.47)	1.12 (0.82–1.43)	1.31 (0.98–1.64)	1.68 (1.30–2.05)	1.55 (1.19–1.91)	1.31 (0.98–1.64)	1.16 (0.85–1.47)
Central-West	Both sexes	3.38 (2.79–3.98)	3.59 (2.99–4.19)	3.30 (2.73–3.87)	3.38 (2.81–3.96)	3.56 (2.98–4.14)	2.95 (2.43–3.46)	3.73 (3.15–4.31)	3.28 (2.75–3.82)	3.39 (2.86–3.93)	3.72 (3.16–4.28)	3.30 (2.78–3.82)	3.28 (2.77–3.80)	3.81 (3.26–4.36)	3.14 (2.65–3.63)	3.37 (2.86–3.88)	3.12 (2.64–3.60)	3.11 (2.63–3.59)	3.24 (2.75–3.72)	3.38 (2.89–3.87)	2.60 (2.17–3.03)	3.55 (3.05–4.05)	3.89 (3.37–4.42)	3.78 (3.26–4.30)	3.78 (3.26–4.30)	3.36 (2.87–3.85)	2.86 (2.41–3.31)
	Male	4.71 (3.72–5.70)	5.00 (3.99–6.01)	4.68 (3.72–5.64)	5.19 (4.18–6.20)	4.69 (3.74–5.63)	4.04 (3.18–4.89)	5.25 (4.27–6.24)	4.84 (3.92–5.76)	4.65 (3.75–5.55)	5.52 (4.55–6.49)	5.54 (4.58–6.50)	5.02 (4.11–5.92)	6.01 (5.03–6.99)	4.70 (3.85–5.56)	4.96 (4.09–5.84)	4.67 (3.84–5.50)	4.47 (3.65–5.28)	5.02 (4.16–5.88)	5.29 (4.41–6.16)	3.93 (3.18–4.69)	4.93 (4.09–5.77)	5.90 (4.99–6.82)	5.13 (4.27–5.98)	5.76 (4.85–6.66)	5.23 (4.37–6.10)	4.37 (3.58–5.16)
	Female	2.10 (1.44–2.76)	2.23 (1.56–2.90)	1.97 (1.34–2.60)	1.64 (1.08–2.20)	2.47 (1.79–3.15)	1.89 (1.30–2.48)	2.26 (1.62–2.90)	1.78 (1.22–2.34)	2.18 (1.57–2.79)	1.98 (1.41–2.56)	1.14 (0.71–1.57)	1.60 (1.09–2.12)	1.68 (1.16–2.20)	1.63 (1.12–2.13)	1.83 (1.31–2.36)	1.63 (1.14–2.12)	1.80 (1.29–2.32)	1.51 (1.04–1.98)	1.53 (1.06–2.00)	1.32 (0.88–1.75)	2.21 (1.65–2.77)	1.95 (1.42–2.48)	2.48 (1.88–3.08)	1.87 (1.35–2.38)	1.54 (1.07–2.02)	1.40 (0.95–1.85)

Appendix E

**Table 9:** Observed and expected age- and sex-standardized acute myocardial infarction mortality during and after the COVID-19 pandemic, adults aged 20-39 years, Brazil, 2020–2025. Each slope was tested against the null of no change with a two-sided Student's t test on the regression coefficient (t = β/SE(β)). Two-sided p < 0.05 was considered statistically significant. A positive difference means the observed rate exceeded the rate the pre-pandemic trend predicted for that year. The sensitivity of the trend estimate to these years is given in Appendix F. APC, annual percent change Sources: Deaths, Mortality Information System (SIM), Brazilian Ministry of Health [17]; annual population denominators, IBGE population projections, 2024 revision [19]; standard population, 2022 Brazilian Demographic Census [20]

Year	Expected rate	Observed rate	Difference, %
2020	3.71	3.62	−2.6
2021	3.71	3.86	+4.2
2022	3.70	3.93	+6.1
2023	3.70	3.86	+4.4
2024	3.70	3.81	+3.0
2025	3.70	3.76	+1.6

Appendix F 

**Table 10:** Sensitivity of the national trend estimate to exclusion of the pandemic years and of the provisional final year, adults aged 20-39 years, Brazil, 2000–2025. Each slope was tested against the null of no change with a two-sided Student's t test on the regression coefficient (t = β/SE(β)). Two-sided p < 0.05 was considered statistically significant. Joinpoint models have df = 22 and single-segment models df = 24 (df = 22 when two years are excluded). Excluding the two pandemic years left both the joinpoint locations and the summary estimate essentially unchanged, indicating that the overall trend is not an artifact of the pandemic period. AAPC, average annual percent change; APC, annual percent change Sources: Deaths, Mortality Information System (SIM), Brazilian Ministry of Health [17]; annual population denominators, IBGE population projections, 2024 revision [19]; standard population, 2022 Brazilian Demographic Census [20]

Analysis	Joinpoints	Estimate,	95% CI	t	p	Estimate type
		%/year				
Full period (2000-2025)	2005; 2009	−0.23	−0.50 to +0.04	−1.78	0.088	AAPC
Excluding 2020-2021	2005; 2009	−0.22	−0.50 to +0.06	−1.67	0.11	AAPC
Excluding 2025 (provisional)	2005; 2009	−0.24	−0.53 to +0.05	−1.69	0.105	AAPC
Single-segment, full period (2000-2025)	—	0.05	−0.19 to +0.28	0.42	0.68	APC
Single-segment, excluding 2020-2021	—	0.06	−0.20 to +0.31	0.46	0.652	APC
Single-segment, excluding 2025 (provisional)	—	0.05	−0.20 to +0.31	0.44	0.668	APC

Appendix G

**Table 11:** Stability of the selected joinpoint model under parametric bootstrap, national series, Brazil, 2000–2025. No hypothesis test is reported in this table: the quantities are frequencies across bootstrap replicates, not test statistics. The joinpoint years varied widely across replicates and should be read as approximate, whereas the absence of change in the final segment was reproduced in the large majority of replicates. Sources: Deaths, Mortality Information System (SIM), Brazilian Ministry of Health [17]; annual population denominators, IBGE population projections, 2024 revision [19]; standard population, 2022 Brazilian Demographic Census [20]

Quantity	Value
Two joinpoints selected	1,189 of 2,000 replicates (59.5%)
One joinpoint selected	579 (28.9%)
No joinpoint selected	232 (11.6%)
Second joinpoint, median (95% range)	2009 (2008 to 2019)
Final segment not significant at p < 0.05	1,787 of 2,000 replicates (89.3%)

Appendix H 

**Table 12:** Tipping-point bias analysis: change in completeness of cause-of-death certification that would be required for each finding to be entirely artifactual, Brazil, 2000–2025 No hypothesis test is reported in this table: the thresholds are deterministic bias quantities, not test statistics. The table reads as follows: for the rise at ages 20–24 years to be entirely an artifact of improving death certification, completeness would have to have increased by 120% over the period. The differential rows are the more stringent test, since adjacent age bands and the two sexes are certified by the same services in the same municipalities under the same coding rules. The redistribution these thresholds anticipate was carried out and is reported in Appendices I, J. APC, annual percent change Sources: Deaths, Mortality Information System (SIM), Brazilian Ministry of Health [17]; annual population denominators, IBGE population projections, 2024 revision [19]; standard population, 2022 Brazilian Demographic Census [20]

Finding	APC, %/year	Required change in certification completeness, 2000–2025
Ages 20–24 years, rise	3.21	120%
Ages 25–29 years, rise	1.73	54%
20–24 vs 35–39 divergence	—	+165% differential
North, rise	1.32	39%
South, decline	−2.28	−44%
Men vs women divergence	—	+24% differential

Appendix I

**Table 13:** Share of all deaths at ages 20-39 years carried by ill-defined and nonspecific cause-of-death codes, by macroregion, Brazil, 2000 and 2025. Ill-defined and nonspecific codes are R00–R99 together with the nonspecific cardiovascular codes I46, I50, I51 and I99 [18]. Data are presented as the percentage of all deaths at ages 20-39 years in that region and year carried by those codes. This table sets the size of the misclassification problem; its correction is reported in Appendix J. No hypothesis test is reported in this table: the values are observed proportions. The fall was steepest in the North and Northeast, the regions where the recorded increase in acute myocardial infarction mortality was largest, which is why redistribution was carried out rather than assumed to be immaterial. Source: Deaths, Mortality Information System (SIM), Brazilian Ministry of Health [17]; cause of death coded to ICD-10 [18]

Macroregion	2000, %	2025, %
Brazil	10.1	6.3
North	16.5	6.0
Northeast	16.1	5.0
Southeast	8.2	8.0
South	5.0	4.9
Central-West	6.7	6.0

Appendix J

**Table 14:** Trend estimates before and after proportional redistribution of ill-defined and nonspecific cause-of-death codes, by macroregion, sex and age band, Brazil, 2000–2025. Estimates come from the same single-segment weighted log-linear model as Table 2 and were tested the same way (two-sided Student's t test on the regression coefficient, df = 24; p < 0.05 considered significant). Redistribution raised the death count entering the rates from 59,480 to 64,309 (+8.1%). Because the nonspecific cardiovascular codes were redistributed across all causes rather than within circulatory disease alone, the correction is conservative for that group. Confidence intervals on the corrected estimates treat redistributed counts as observed and therefore do not incorporate uncertainty in the redistribution itself. APC, annual percent change Sources: Deaths, Mortality Information System (SIM), Brazilian Ministry of Health [17]; annual population denominators, IBGE population projections, 2024 revision [19]; standard population, 2022 Brazilian Demographic Census [20]

Stratum	APC observed	95% CI	p	APC corrected	95% CI	p
Brazil	0.05	−0.19 to +0.28	0.68	−0.18	−0.42 to +0.07	0.15
North	1.32	+0.67 to +1.97	<0.001	0.86	+0.30 to +1.42	0.004
Northeast	0.43	−0.17 to +1.04	0.149	−0.00	−0.46 to +0.46	0.988
Southeast	0.26	−0.20 to +0.73	0.252	0.2	−0.28 to +0.68	0.398
South	−2.28	−2.72 to −1.85	<0.001	−2.34	−2.77 to −1.90	<0.001
Central-West	−0.03	−0.56 to +0.50	0.914	−0.16	−0.67 to +0.36	0.528
Brazil, men	0.29	+0.06 to +0.52	0.016	0.11	−0.12 to +0.35	0.341
Brazil, women	−0.57	−0.86 to −0.28	<0.001	−0.90	−1.21 to −0.59	<0.001

Appendix K 

**Table 15:** Serial-correlation diagnostics and inference robust to serial correlation, by stratum, Brazil, 2000–2025. First-order dependence was detected at p < 0.05 in 11 of 22 single-segment summaries but in only 4 of 22 selected joinpoint models, the median Durbin-Watson statistic rising from 1.20 to 1.89; the segmented specification therefore absorbs most of the temporal dependence. Newey-West standard errors were 0.78 to 1.60 times the model-based values (median 1.25). Two-sided p < 0.05 was considered statistically significant. APC, annual percent change; DW, Durbin-Watson statistic; LM, Lagrange multiplier; N-W, Newey-West; ρ, first-order autocorrelation coefficient of the weighted residuals of the selected model Sources: Deaths, Mortality Information System (SIM), Brazilian Ministry of Health [17]; annual population denominators, IBGE population projections, 2024 revision [19]; standard population, 2022 Brazilian Demographic Census [20]

Series	DW single	DW selected	ρ	p (LM)	APC, %/year	95% CI, model-based	95% CI, Newey-West	p model	p N-W
Brazil, both sexes	0.92	1.13	0.44	0.029	+0.05	−0.19 to +0.28	−0.24 to +0.34	0.680	0.736
Brazil, male	0.92	1.08	0.46	0.021	+0.29	+0.06 to +0.52	+0.03 to +0.55	0.016	0.030
Brazil, female	1.31	1.80	0.06	0.761	−0.57	−0.86 to −0.28	−0.94 to −0.20	< 0.001	0.004
North, both sexes	1.65	2.80	−0.42	0.034	+1.32	+0.67 to +1.97	+0.57 to +2.08	< 0.001	0.001
North, male	1.30	2.54	−0.29	0.133	+1.73	+0.99 to +2.47	+0.79 to +2.67	< 0.001	< 0.001
North, female	2.03	2.36	−0.19	0.337	+0.31	−0.57 to +1.20	−0.45 to +1.08	0.473	0.407
Northeast, both sexes	0.38	1.42	0.25	0.188	+0.43	−0.17 to +1.04	−0.47 to +1.35	0.149	0.333
Northeast, male	0.40	1.50	0.23	0.245	+0.75	+0.06 to +1.44	−0.28 to +1.79	0.033	0.146
Northeast, female	1.09	2.42	−0.23	0.235	−0.34	−0.87 to +0.20	−1.03 to +0.36	0.206	0.328
Southeast, both sexes	0.49	1.19	0.40	0.045	+0.26	−0.20 to +0.73	−0.48 to +1.01	0.252	0.470
Southeast, male	0.53	1.22	0.39	0.052	+0.48	−0.01 to +0.96	−0.27 to +1.23	0.054	0.202
Southeast, female	1.06	2.05	−0.07	0.726	−0.30	−0.81 to +0.21	−1.06 to +0.47	0.241	0.430
South, both sexes	0.93	1.69	0.15	0.457	−2.28	−2.72 to −1.85	−2.95 to −1.61	< 0.001	< 0.001
South, male	1.48	2.57	−0.29	0.152	−2.19	−2.68 to −1.70	−2.84 to −1.54	< 0.001	< 0.001
South, female	1.78	1.78	0.07	0.712	−2.49	−3.28 to −1.69	−3.32 to −1.66	< 0.001	< 0.001
Central-West, both sexes	1.81	2.18	−0.13	0.535	−0.03	−0.56 to +0.50	−0.58 to +0.53	0.914	0.918
Central-West, male	1.98	1.98	−0.05	0.828	+0.22	−0.37 to +0.80	−0.24 to +0.67	0.453	0.338
Central-West, female	1.64	2.37	−0.20	0.330	−0.68	−1.66 to +0.32	−1.79 to +0.44	0.171	0.224
Brazil, 20–24 years	0.77	2.06	−0.04	0.823	+3.21	+2.41 to +4.01	+2.22 to +4.20	< 0.001	< 0.001
Brazil, 25–29 years	1.52	1.99	−0.05	0.802	+1.73	+1.39 to +2.08	+1.45 to +2.02	< 0.001	< 0.001
Brazil, 30–34 years	1.52	1.52	0.18	0.332	+0.26	−0.07 to +0.59	−0.06 to +0.57	0.123	0.105
Brazil, 35–39 years	1.07	1.34	0.32	0.107	−0.73	−0.99 to −0.46	−1.06 to −0.39	< 0.001	< 0.001

Appendix L 

**Table 16:** Acute myocardial infarction mortality trends in age ranges outside the study population, shown for context, Brazil, 2000–2025. Each slope was tested against the null of no change with a two-sided Student's t test on the regression coefficient (t = β/SE(β)). Two-sided p < 0.05 was considered statistically significant. The 65-and-over range uses an 80+ terminal band because the death series is not published in finer detail above that age. AAPC, average annual percent change Sources: Deaths, Mortality Information System (SIM), Brazilian Ministry of Health [17]; annual population denominators, IBGE population projections, 2024 revision [19]; standard population, 2022 Brazilian Demographic Census [20]

Age range	Deaths, n	Rate	Rate	Change,	AAPC, %/year (95% CI)	t	p
		2000	2025	%			
20–39 years (study population)	59,480	4.00	3.76	−6.2	−0.23 (−0.50 to +0.04)	−1.78	0.088
40–44 years	59,274	21.46	13.91	−35.2	−1.68 (−1.90 to −1.46)	−15.77	<0.001
20–44 years (composite)	118,754	7.57	5.83	−23.0	−1.00 (−1.21 to −0.79)	−9.97	<0.001
40–64 years	727,093	66.23	42.96	−35.1	−1.61 (−1.77 to −1.46)	−20.95	<0.001
65 years and over	1,330,132	327.80	245.67	−25.1	−1.06 (−1.20 to −0.91)	−15.09	<0.001
